# Higher BMP/Smad sensitivity of tendon-derived stem cells (TDSCs) isolated from the collagenase-induced tendon injury model: possible mechanism for their altered fate *in vitro*

**DOI:** 10.1186/1471-2474-14-248

**Published:** 2013-08-21

**Authors:** Pauline Po Yee Lui, Yin Mei Wong

**Affiliations:** 1 Headquarter, Hosptial Authority, 9/F, Rumsey Street Multi-Storey Carpark Building, Sheung Wan, Hong Kong SAR, China; 2 Department of Orthopaedics and Traumatology, Faculty of Medicine, The Chinese University of Hong Kong, Hong Kong SAR, China; 3 The Hong Kong Jockey Club Sports Medicine and Health Sciences Centre, Faculty of Medicine, The Chinese University of Hong Kong, Hong Kong SAR, China

**Keywords:** Tendinopathy, Tendon-derived stem/progenitor cells, BMP/Smad signaling, Collagenase-induced tendon injury

## Abstract

**Background:**

Ectopic expression of BMP-2, BMP-4 and BMP-7 was observed in clinical samples of tendinopathy and collagenase-induced (CI) tendon injury rat model. TDSCs isolated from the CI model showed increased non-tenogenic differentiation potential and hence altered fate compared to the TDSCs isolated from the healthy animals (HT) but the mechanism is unclear. We hypothesized that sensitization of the BMP/Smad pathway in TDSCs (CI) might account for this difference. This study aimed to compare the activation state of the BMP/Smad pathway at basal level and upon BMP-2 stimulation in TDSCs (CI) and TDSCs (HT).

**Methods:**

Collagenase or saline was injected into the patellar tendon of rats for 2 weeks. TDSCs (CI) and TDSCs (HT) were then isolated from the patellar tendon. The mRNA and protein expression of BMPs and BMP receptors in TDSCs (CI) and TDSCs (HT) were analysed. TDSCs from both sources were treated with rhBMP-2 and the expression of phosphorylated and total Smad1/5/8 was examined.

**Results:**

Except for the mRNA levels of *Bmp7* and *Bmpr2*, there were significant higher mRNA and protein expression of BMPs and BMP receptors in TDSCs (CI) compared to TDSCs (HT). TDSCs (CI) showed higher basal expression of total Smad1/5/8 but similar basal level of phosphorylated Smad1/5/8 compared to TDSCs (HT). TDSCs (CI) exhibited higher total and phosphorylated Smad1/5/8 upon BMP-2 stimulation.

**Conclusions:**

The sensitization of the BMP/Smad pathway in TDSCs (CI) might account for their higher non-tenogenic differentiation potential and hence altered fate. It also provided further support of BMPs and the BMP/Smad signaling pathway in the pathogenesis of tendinopathy.

## Background

Tendinopathy is a chronic painful tendon disorder that is common among athletes and middle-aged people with repetitive tendon overuse. Histopathologically, tendinopathy is characterized by an increase of cellularity [1-3], vascularity [1], glycosaminoglycan deposition [1,2] and loss of matrix organization [1,4]. Tissue metaplasia, with the presence of chondrocyte phenotype [5] and occasional fatty and bony deposits [2,4,5], is observed. The pathogenesis of tendinopathy remains unclear. As a result, only symptomatic treatments are currently provided with limited success [6,7]. The cells isolated from the tendinopathic tissue showed high metabolic activity [3]. Therefore, failed healing, rather than degeneration, is suggested as the pathogenesis of tendinopathy [8].

Ectopic expression of BMP-2, -4 and -7 were observed in clinical samples of tendinopathy [9,10] and collagenase-induced (CI) tendon injury rat model which showed failed healing and ectopic chondro-ossification in tendons [11]. These BMPs were reported to promote osteogenic, chondrogenic and adipogenic differentiation of mesenchymal stem cells (MSCs) including tendon-derived stem cells (TDSCs) isolated from healthy tendons *in vitro*[12-16]. Tendinopathic Achilles tendons showed higher mRNA expression of chondro-osteogenic markers compared to healthy tendons [17]. Consistent with this previous finding [17], TDSCs isolated from the CI model (TDSCs (CI)) showed altered fate, with higher chondro-osteogenic differentiation potential but lower tenogenic marker expression compared to TDSCs isolated from the healthy animals (TDSCs (HT)) [18]. However, the mechanism of altered fate of TDSCs(CI) is unclear. We hypothesized that the increased sensitivity of the BMP/Smad signaling pathway in TDSCs (CI) might account for their higher non-tenogenic differentiation potential and hence altered fate. This study therefore aimed to compare the activation state of the BMP/Smad pathway at basal level and upon BMP-2 stimulation in TDSCs isolated from CI model and healthy animals. Results from this study would provide novel insight of the pathogenic mechanism of tendinopathy.

## Methods

### Collagenase-induced tendon injury model

This study was approved by the Animal Research Ethics Committee of the authors’ institution (Ref no: 10/010/GRF). Twelve male Sprague Dawley rats, (6 weeks, weight 150-220 grams) were used. The procedures have been well-established and the histopathological changes were highly reproducible [19]. After anesthesia with 2.5% pentobarbital (4.5 mg/kg body weight), hairs over the lower limb were shaved. The patellar tendon was located by positioning the knee at 90°. Twenty microliters (0.015 mg/μl in 0.9% saline, i.e. 0.3 mg) of bacterial collagenase I (Sigma-Aldrich, St Louis, MO, USA) (CI group) (n = 6) or saline (HT group) (n = 6) were injected into both patellar tendons (i.e. both tendons were injected with collagenase or both tendons were injected with saline) of each rat intratendinously with a 30 G needle. Free cage activity was allowed after injection. At week 2 after injection, rats were sacrificed and both patellar tendons of each rat were harvested and pooled together for the isolation of TDSCs (HT) or TDSCs (CI) [19]. Week 2 was chosen for the isolation of TDSCs (CI) when the direct effect of collagenase subsided and tendon healing with increase in cell proliferation occurred while no chondrocyte-like cells were observed at this time point [19].

### Isolation and culture of rat TDSCs

The procedures for the isolation of TDSCs from the mid-substance of collagenase-injured and healthy patellar tendons have been established [20]. Briefly, after euthanasia, the mid-substances of both patellar tendons were excised. Care was taken that only the mid-substance of patellar tendon tissue, but not the tissue at the tendon-bone junction, was collected. The peritenon was carefully removed and the tissue was stored in sterile phosphate-buffered saline (PBS). The tissue was minced, digested with type I collagenase (3 mg/ml; Sigma-Aldrich, St Louis, MO, USA) and passed through a 70 μm cell strainer (Becton Dickinson, Franklin Lakes, USA) to yield a single-cell suspension. The released cells were washed in PBS and resuspended in low glucose Dulbecco’s Modified Eagle Medium (LG-DMEM) (Gibco BRL; Life Technologies, Invitrogen, Carlsbad, CA, USA), 10% fetal bovine serum (FBS), 50 μg/ml penicillin, 50 μg/ml streptomycin 100 μg/ml neomycin (complete culture medium) (all from Invitrogen corporation, Carlsbad, USA). The isolated nucleated cells were plated at an optimal low density (50 cells/cm^2^) for the isolation of TDSCs from rat patellar tendon and cultured at 37°C, 5% CO_2_ to form colonies. At day 2 after initial plating, the cells were washed twice with PBS to remove the non-adherent cells. At day 7-10, they were trypsinized and mixed together as passage 0 (P0). TDSCs were subcultured when they reached 80-90% confluence. The stem cell-related surface marker expression (including CD44, CD90 and CD73), clonogenicity and multi-lineage differentiation potential of the isolated nucleated cells from the CI animal model and healthy animals were confirmed as described previously before being used for the experiments in this study [18]. TDSCs (CI) and TDSCs (HT) at passage 5 were used for all the experiments.

### Study design

TDSCs isolated from both sources were plated at 4000 cell/cm^2^ in 100-mm tissue culture dish and cultured in low glucose Dulbecco’s Modified Eagle Medium (LG-DMEM) (Gibco) supplemented with 10% fetal bovine serum (FBS), 50 μg/ml penicillin, 50 μg/ml streptomycin and 100 μg/ml neomycin (complete culture medium) (all from Invitrogen corporation, Carlsbad, USA) at 37°C, 5% CO_2_ until confluence. The cells were then subjected to mRNA and protein analysis of expression of BMPs (BMP-2, BMP-4, BMP-7) and BMP receptors (BMPR-IA, BMPR-IB, BMPR-II) using qRT-PCR and Western blotting (WB), respectively. To investigate the response of both cell types to BMP-2 stimulation, TDSCs (CI) and TDSCs (HT) isolated from each of three rats were plated at 4000 cell/cm^2^ in 6-wells plate and cultured in complete medium until the cells reached 80% confluence for immunocytochemical staining (ICC) and confluence for WB. They were then treated with rhBMP-2 (100 ng/ml) (Wyeth, Cambridge, MA, USA) in complete medium for 0, 15, 30 and 60 minutes at 37°C, 5% CO_2_. Time series data of TDSC (CI) and TDSC (HI) of each of 4 rats / group was obtained. The nuclear translocation of the phosphorylated form of Smad 1/5/8 (pSmad 1/5/8) was examined by ICC while the expression of the pSmad 1/5/8 and total Smad 1/5/8 was examined by WB. BMP-2 at 100 ng/ml was used in this study based on our previous study testing the effect of different concentrations of BMP-2 (0, 50, 100, 250, 500 and 1000 ng/ml) and 100 ng/ml was the lowest dose that induced the osteogenic differentiation of TDSCs (unpublished results). BMP-2 at 100 ng/ml promoted non-tenogenic (osteo-, chondro- and adiop- genic) differentiation, increased proteoglycan production but inhibited tendon-related marker expression in TDSCs [13]. This same dose (100 ng/ml) was also used in a previous study investigating the effect of BMP-2 on the osteogenic response as well as the expression and translocation of pSmad 1/5/8 in tendon stem / progenitor cells [21].

### Quantitative real-time reverse transcription-polymerase chain reaction (qRT-PCR)

Cells were harvested and homogenized for RNA extraction with Rneasy mini kit (Qiagen, Germany). The mRNA was reverse-transcribed to cDNA by the First Strand cDNA kit (Promega, Madison, WI, USA). 5 μl of total cDNA of each sample was amplified in final volume of 25 μl of reaction mixture containing Platinum® SYBR® Green qPCR SuperMix-UDG ready-to-use reaction cocktail and specific primers for *Bmp2*, *Bmp4*, *Bmp7*, *Bmpr1a*, *Bmpr1b*, *Bmpr2* or *β-actin* using the ABI StepOne Plus system (all from Applied Biosystems, CA, USA) (Table 1). Cycling conditions were: denaturation at 95°C for 10 minutes, 45 cycles at 95°C for 20 seconds, optimal annealing temperature (Table 1) for 20 seconds, 72°C for 30 seconds and finally at 60-95°C with a heating rate of 0.1°C/second. The expression of target gene was normalized to that of *β-actin* gene. Relative gene expression was calculated with the 2^-△CT^ formula. The mRNA expression of BMPs and BMP receptors were the results of 6 rats from each group.

**Table 1:** The primer sequence, product size and annealing temperature of target genes for qRT-PCR

**Gene**	**Primer nucleotide sequence**	**Product size (bp)**	**Annealing temperature**	**Accession no.**
*β-actin*	5′-ATCGTGGGCCGCCCTAGGCA-3′ (forward)	243	52	NM_031144
	5′-TGGCCTTAGGGTTCAGAGGGG-3′ (reverse)			
*Bmp2*	5′-TAGTGACTTTTGGCCACGACG-3′ (forward)	81	58	NM_017178
	5′-GCTTCCGCTGTTTGTGTTTG-3′ (reverse)			
*Bmp4*	5′-CATGGCTCGCGCCTCCTAGC-3′ (forward)	184	58	NM_012827
	5′- ATTCCGAGCGACGCACTGCC-3′ (reverse)			
*Bmp7*	5′- CAACCTAGTGGAGCACGACAAGGA-3′ (reverse)	213	60	NM_001191856
	5′- AGGTCGGACTCCCTGCCTGAGT-3′ (reverse)			
*Bmpr1a*	5′-GCCACCCTGGACACCAGAGC-3′ (forward)	101	60	NM_030849
	5′-GCAGGCTTGCCTTGCGTG-3′ (reverse)			
*Bmpr1b*	5′-CACCACGGAGGAAGCCAGC-3′ (forward)	239	60	NM_001024259
	5′-GGCACAGGCCGCTGACAGAC-3′ (reverse)			
*Bmpr2*	5′-GGTGCTGGTCTCACATTG-3′ (forward)	180	58	NM_080407
	5′-GAGGCGGACTGAGTGGTG-3′ (reverse)			

### Western blotting (WB)

The cells were lysed and the concentration of total soluble protein was measured by BCA protein assay (Thermo Scientific, Rockford, IL). 50 μg (for BMPs and BMP receptors) or 20 μg (for pSmad 1/5/8 and total Smad 1/5/8) of protein was denatured, fractionated by electrophoresis on 12% (w/v) sodium dodecyl sulfate (SDS)-polyacrylamide gel and then electrophoretically transferred onto nitrocellulose membrane (Pall, Ann Arbor, MI). The membrane was blocked with 5% (w/v) non-fat dry milk in TBST solution (25 mM Trizma base (3.025 g), 125 mM NaCl (7.3 g), and 1 mL Tween-20, pH 7.6) and then incubated with primary antibody against BMP-2 (1:1000), BMP-4 (1:1000), BMP-7 (1:1000) (all from Abcam, Cambridge, UK), BMPR-IA (1: 500), BMPR-IB (1:200) (both from Santa Cruz Biotechnology, Santa Cruz, CA, USA), BMPR-II (1: 500; BD BioSciences, San Jose, California, USA), total Smad 1/5/8 (1:1000; Abcam, Cambridge, UK), pSmad 1/5/8 (1:1000; Cell Signaling, Danvers, MA, USA) or β-actin antibody (1:1000, Santa Cruz Biotechnology, Santa Cruz, CA, USA). After washing, the membrane was incubated with horseradish peroxidase (HRP)-conjugated anti-goat secondary antibody (1:1000; Santa Cruz Biotechnology, Santa Cruz, CA, USA), HRP-conjugated anti-mouse secondary antibody (1:1000) or HRP-conjugated anti-rabbit secondary antibody (1:1000) (both from Merck Millipore, Darmstadt, Germany). Immunoreactive bands were detected by ECL reagents (Amersham Bioscience, Little Chalfont, UK). The same batch of samples was used for studying the expression of BMPs and BMPRs. Except for BMP-2, all the proteins used for the detection of BMPs and BMP receptors were loaded and ran within one week. Semi-quantitative image analyses of the protein expression of BMPs, BMP receptors, total Smad 1/5/8 and pSmad 1/5/8 were performed using the Adobe Photoshop software (Adobe Systems Incorporated, CA, USA, version 10.0). After thresholding, the region of interest (ROI) was selected by applying a rectangular box with size that was wide enough to cover the largest band among protein samples and with minimum height possible. The mean expression level of the target protein relative to β-actin was presented. As the expression of BMP-2 was studied at a different time compared to other BMPs and BMP receptors, different β-actin bands were used for normalization. Different β-actin bands of each independent experiment were used for normalization for the expression of total Smad 1/5/8 and pSmad 1/5/8. The Western analyses were the results of 3-4 rats from each group.

### Immunocytochemical staining (ICC)

ICC was performed using UltraVision Quanto Detection System (Thermo Scientific, Kalamazoo, MI, USA). Briefly, the cells were fixed in 70% ethanol, quenched with 3% H_2_O_2_ in methanol for 20 minutes, blocked with Ultra V Block and incubated with rabbit anti-human pSmad 1/5/8 cross-reacted with rat pSmad 1/5/8 (1:200, Cell Signaling, Danvers, MA, USA) overnight at 4°C. Primary antibody was replaced with blocking solution in the negative controls. After that, the cells were incubated with Primary Antibody Amplifier Quanto and then HRP Polymer Quanto for 10 minutes each at room temperature. Signal was visualized with DAB Quanto Chromogen/Substrate complex. The cells were rinsed in distilled water, counterstained in Harris hematoxylin, dehydrated through graded alcohol, and mounted with p-xylene-bis-pyridinium bromide (DPX) (Sigma Aldrich, St Louis, MO, USA). The cells were examined under light microscopy (DMRXA2, Leica Microsystems, Wetzlar GmbH, Germany). Representative results from 3 experiments were reported.

### Data analysis

Quantitative and semi-quantitative data was shown in boxplots or time curve with mean and standard error (SE). For the boxplot, the lower, middle and upper boundaries of the box showed the 25^th^, 50^th^ and 75^th^ percentile of the dataset. Observation with value that was more than 3 box-length from the upper or lower edge of the box was shown as extreme value (*) if existed. Observation with value that was between 1.5 to 3 box-length from the upper or lower edge of the box was shown as outlier (o) if existed. The largest and smallest observations in the dataset that were not outliers or extreme values were shown as whiskers. If there were no outliers and extreme values, the whiskers represented the maximum and the minimum observations of the dataset. The comparison of mRNA and protein expression of BMPs and BMP receptors between the TDSCs (CI) group and the TDSCs (HT) group was done using Mann-Whitney U test. The comparison of the time series data of total Smad 1/5/8 and pSamd 1/5/8 between the TDSCs (CI) group and the TDSCs (HT) group was done using ANOVA fore repeated measures with time as the within-subjects factor and treatment group as the between-subjects factor. All the data analysis was done using SPSS (SPSS Inc, Chicago, IL, version 16.0). p < 0.050 was regarded as statistically significant.

## Results

**Figure 1:**
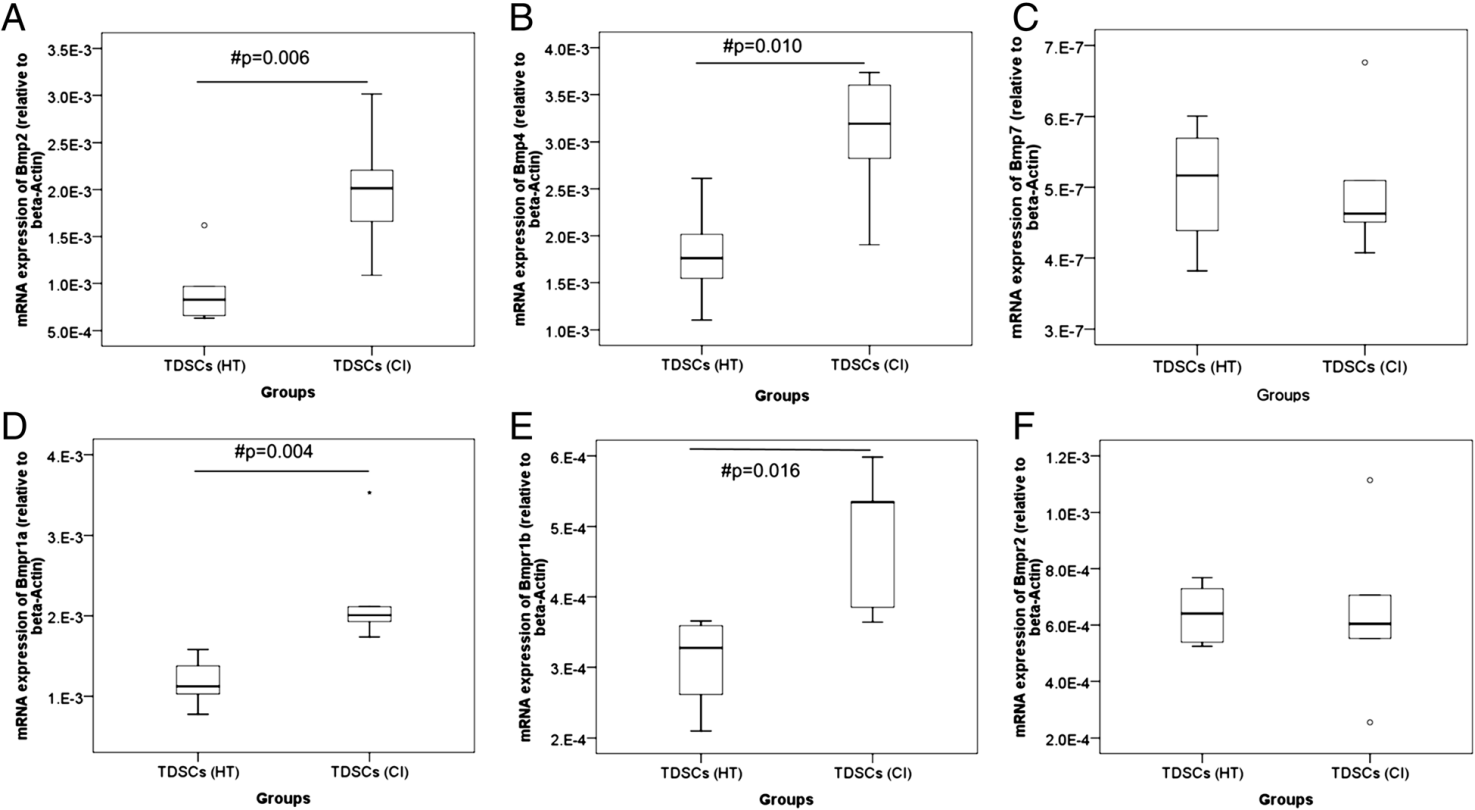
**mRNA expression of BMPs and BMP receptors in TDSCs (HT) and TDSCs (CI).** Boxplots showing the mRNA expression of **(A)***Bmp2*, **(B)***Bmp4*; **(C)***Bmp7*, **(D)***Bmpr1a*; **(E)***Bmpr1b* and **(F)***Bmpr2* in TDSCs (HT) and TDSCs (CI). # indicates p < 0.050; “o” and “*” represented outlier and extreme value, respectively, in the study.

**Figure 2:**
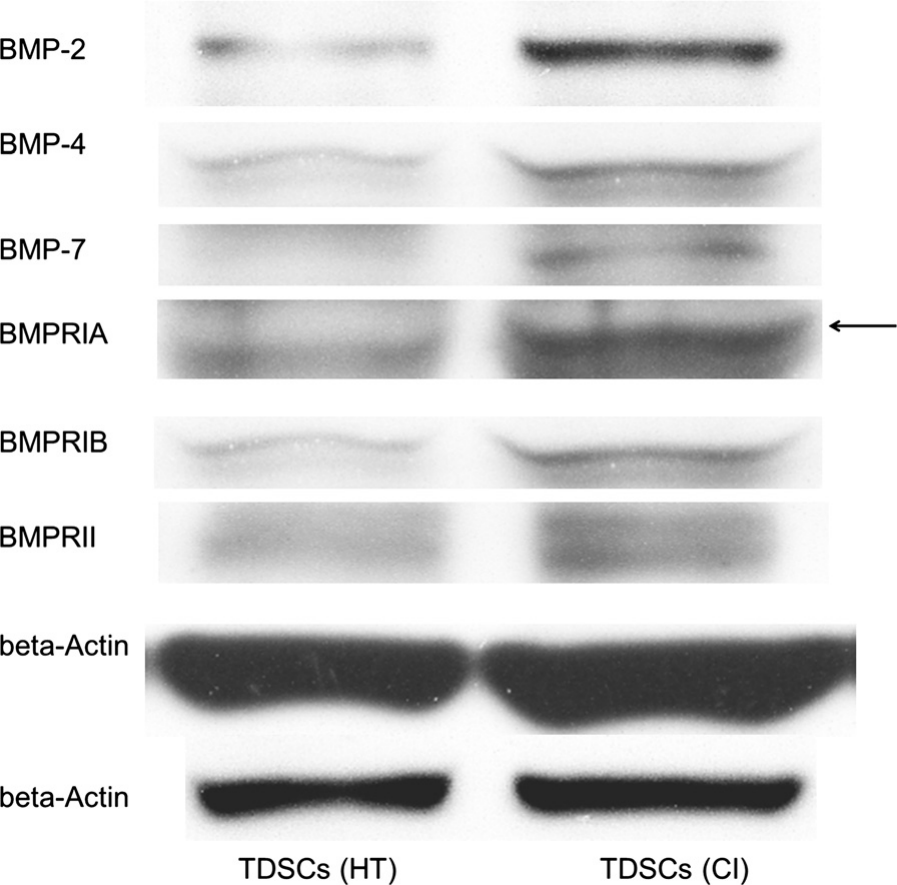
**Protein expression of BMPs and BMP receptors in TDSCs (HT) and TDSCs (CI).** Photograph showing the protein expression of BMPs and BMP receptors in TDSCs (HT) and TDSCs (CI). The β-actin bands on the lower row were the loading references for BMP-2 while the β-actin bands on the upper row were the loading references for the other markers.

**Figure 3:**
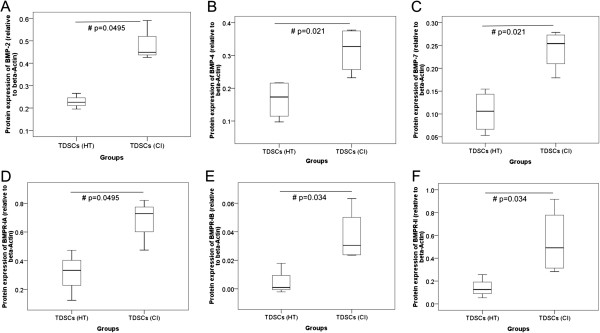
**Semi-quantitative analysis of BMPs and BMP receptors in TDSCs (HT) and TDSCs (CI).** Boxplots showing the semi-quantitative analysis of the protein band intensity relative to β-actin for **(A)** BMP-2, **(B)** BMP-4, **(C)** BMP-7, **(D)** BMPR-IA, **(E)** BMPR-IB and **(F)** BMPR-II in TDSCs (HT) and TDSCs (CI). # indicates p < 0.050.

**Figure 4:**
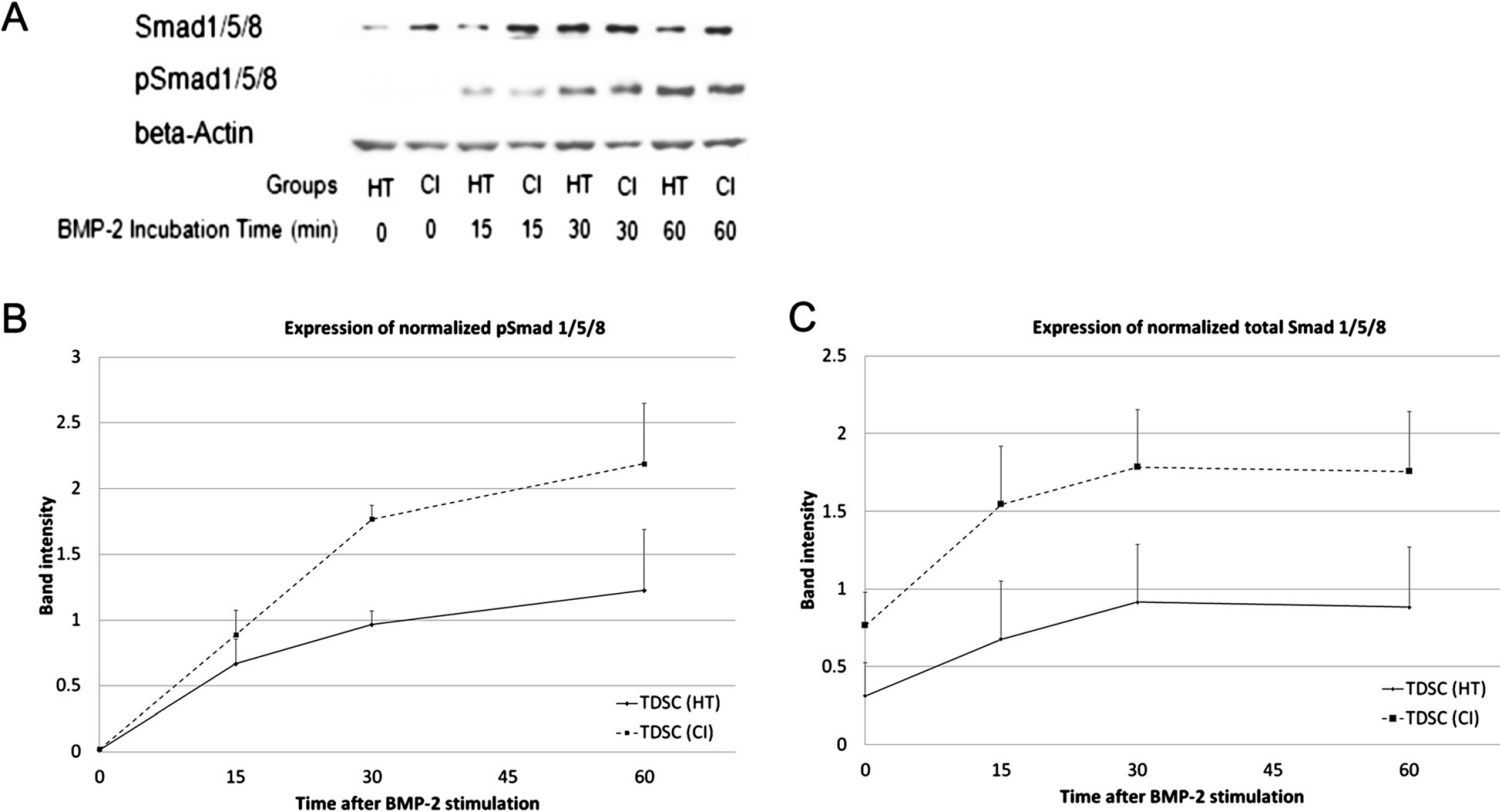
**Total and phosphorylated Smad 1/5/8 in TDSCs (HT) and TDSCs (CI) after BMP-2 stimulation. (A)** Photograph showing the protein expression of total Smad 1/5/8 and pSmad 1/5/8 in TDSCs (HT) and TDSCs (CI) at 0 min, 15 min, 30 min and 60 min after BMP-2 stimulation. β-actin was used as a loading control. Graphs showing the normalized mean and SE of protein band intensities of **(B)** pSmad 1/5/8 and **(C)** total Smad 1/5/8 at 0 min, 15 min, 30 min and 60 min after BMP-2 stimulation.

**Figure 5:**
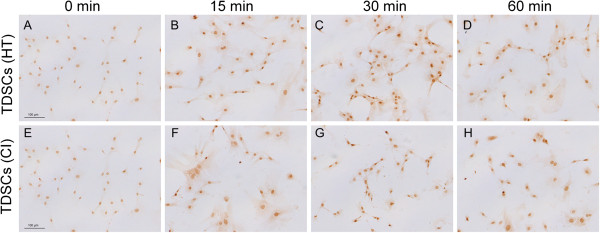
**Nuclear translocation of pSmad 1/5/8.** Photographs showing the expression of pSmad 1/5/8 in **(A**-**D)** TDSCs (HT) and **(E**-**H)** TDSCs (CI) at **(A**, **E)** 0 min, **(B**, **F)** 15 min; **(C**, **G)** 30 min and **(D**, **H)** 60 min after BMP-2 stimulation. Scale bar = 100 μm.

TDSCs (CI) expressed significantly higher mRNA levels of *Bmp2* (2.2 fold, p = 0.006), *Bmp4* (1.7 fold, p = 0.010); *Bmpr1a* (1.9 fold, p = 0.004) and *Bmpr1b* (1.6 fold, p = 0.016) but not *Bmp7* (p = 0.670) and *Bmpr2* (p = 0.873) compared to TDSCs (HT) (Figure 1). TDSCs (CI) also expressed significantly higher protein levels of BMP-2 (2.1 fold, p = 0.0495), BMP-4 (1.9 fold, p = 0.021), BMP-7 (2.3 fold, p = 0.021), BMPR-IA (2.2 fold, p = 0.0495), BMPR-IB (6.6 fold, p = 0.034) and BMPR-II (3.8 fold, p = 0.034) compared to TDSCs (HT) (Figures 2 and 3). There was higher expression of total Smad 1/5/8, but not pSmad 1/5/8 (both nuclear and cytosolic), in TDSCs (CI) compared to TDSCs (HT) (Figure 4A, B, C). Activated pSmad 1/5/8 was mainly located in the cell nucleus (Figure 5A, E). The addition of BMP-2 resulted in the increased expression and translocation of pSmad 1/5/8 to the cell nucleus in both TDSCs (Figure 5B-D, F-H). The expression of total Smad 1/5/8 and pSmad 1/5/8 (both nuclear and cytosolic) increased upon BMP-2 stimulation in both groups (Figure 4A, B, C). There was higher expression of pSmad 1/5/8 (both nuclear and cytosolic) and total Smad in TDSCs (CI) compared to TDSCs (HT) upon BMP-2 stimulation (pSmad 1/5/8: p = 0.065; total Smad: p = 0.043) (Figure 4B, C).

## Discussion

While tissue stem/progenitor cells are essential for maintaining tissue homeostasis and repair, diseases may occur if the control of their renewal and differentiation goes aberrant [22]. We have previously hypothesized that TDSCs might undergo aberrant differentiation to non-tenocyte lineages, contributing to tissue metaplasia and failed healing in tendinopathy [22]. Our recent data supported this claim as TDSCs (CI) showed altered fate, with higher chondro-osteogenic differentiation potential but lower tendon-related marker expression compared to TDSCs (HT), which might contribute to pathological chondro-ossification and failed tendon healing in this animal model [18]. The mechanism(s) that contribute to the altered fate of TDSCs in the CI animal model is unclear. Both mechanical and biological factors might contribute to this. The present study was the first step to understand the mechanisms of altered fate of TDSC (CI) compared to TDSC (HT). In this study, we showed that the increased expression of BMPs and BMP receptors as well as elevated BMP/Smad sensitivity in TDSCs (CI) might contribute to the increased non-tenogenic differentiation potential and hence altered fate of these cells. While we detected increased protein expression of BMP-7 and BMPR-II in TDSCs (CI) compared to TDSCs (HT), we failed to detect increased expression of their corresponding mRNA. It might be due to different half-lives of protein and mRNA.

There was ectopic expression of BMP-2, BMP-4 and BMP-7 in clinical samples of tendinopathy [9,10] and collagenase-induced tendon injury rat model [11]. No expression of BMP-2, BMP-4 and BMP-7 was observed in the intact tendons [9,11]. By exploring the mechanisms of the altered fate of TDSCs (CI), the present study added further support for the role of BMPs and the BMP/Smad signaling pathway in the pathogenesis of tendinopathy. We didn’t examine the effect of BMP-2 on the non-tenogenic differentiation of TDSC (CI) and TDSC (HT) in this study because our previous results have already demonstrated that TDSC (CI) showed higher spontaneous chondro-osteogenesis in basal medium compared to TDSC (HT) [18]. Moreover, BMP-2 alone in basal culture medium was sufficient to induce non-tenogenic differentiation of TDSCs [13]. Similar to our findings, tendon stem / progenitor cells (TSPCs) isolated from disorganized, calcified tendons of biglycan and fibromodulin double knock-out mice were more sensitive to BMP-2 stimulation with increased phosphorylation of Smad 1/5/8 and more abundant nuclear localization of phosphorylated Smad1/5/8 than that in wild-type cells [21]. Fibrodysplasia Ossificans Progressiva (FOP) is characterized by progressive heterotropic ossification. Connective tissue progenitor cells from the discarded primary teeth of patients with FOP showed more rapid differentiation to an osteogenic phenotype as well as higher basal and stimulated BMP signaling compared to those in the control cells [23]. Of note was that both the transgenic animal model [21] and the FOP patients [23] have genetic mutations while the animal model that we used in this study was established using biological agents after birth.

The sources of BMPs after collagenase-induced tendon injury were unknown. The change of tendon loading as a result of tendon overuse has been suggested as one of the etiological factors of tendinopathy [24]. Our previous study showed that TDSCs isolated from the healthy animals produced BMP-2 in response to repetitive cyclic tensile loading [14]. Whether tendon overuse would lead to increased BMP sensitivity of TDSCs as reported in this study and how the increased expression of BMPs and BMP receptors would feedback and modulate the sensitivity of TDSCs to mechanical loading requires further research. Besides mechanical loading, the sources of BMPs might come from other cell types such as tenocytes and/or release from the extracellular matrix after collagenase-induced tendon injury. Further research is needed to confirm our speculation.

This study has some limitations. First, the pathological concentration of BMP-2 in tendinopathy was not known. The dose used in this study was chosen based on our previous study [13] and another report [21]. The use of pathological concentration of BMP-2 in tendinopathy for TDSC stimulation *in vitro* would yield more clinically relevant data. This study was our first step to understand the mechanisms of non-tenogenic differentiation and hence altered fate of TDSCs (CI). A more fundamental question of why TDSCs (CI) have higher BMP and BMPR expression as well as elevated BMP/Smad sensitivity remains unanswered. We did not examine the fate of TDSCs (CI) by inhibiting the BMP/Smad signaling pathway in this study, the results of which would provide further support of BMPs and the BMP/Smad signaling pathway in regulating the fate of TDSCs (CI) and might shed lights on the pathogenesis and treatment of tendinopathy. Further study is required.

## Conclusions

In conclusion, except for the mRNA levels of *Bmp7* and *Bmpr2*, TDSCs isolated from the collagenase-induced patellar tendon injury rat model expressed higher mRNA and protein levels of BMPs (BMP-2, BMP-4, BMP-7) and BMP receptors (BMPR-IA, BMPR-IB, BMPR-II) compared to the TDSCs isolated from the healthy animals. They were also more sensitive to BMP-2 stimulation compared to the TDSCs isolated from the healthy animals. The sensitization of the BMP/Smad pathway might account for the higher non-tenogenic differentiation potential and hence altered fate of TDSCs in the collagenase-induced tendon injury rat model. It also provided further support of BMPs and the BMP/Smad signaling pathway in the pathogenesis of tendinopathy.

## Competing interests

The authors declare that they have no competing interests.

## Authors’ contributions

PPYL conceived of the study, designed the study, analyzed the data and drafted the manuscript. YMW performed the experiments and analyzed the data. Both authors read and approved the final manuscript.

## Pre-publication history

The pre-publication history for this paper can be accessed here:

http://www.biomedcentral.com/1471-2474/14/248/prepub
